# Combined recession-resection in the management of paralytic strabismus: simplification of the complex (a case report)

**DOI:** 10.11604/pamj.2024.48.165.44739

**Published:** 2024-08-09

**Authors:** Kafin Rifqi, Rozalina Loebis

**Affiliations:** 1 Department of Ophthalmology, Faculty of Medicine Airlangga University, Dr. Soetomo General Academic Hospital, Surabaya, Indonesia

**Keywords:** Oculomotor nerve palsy, exotropia, management, good health, case report

## Abstract

Oculomotor nerve palsy presents the greatest challenge in clinical treatment among the other cranial nerves that innervate extraocular muscle. With a complex technique of periosteal fixation using Callahan suture commonly used, a simpler and more effective technique is demanded to be proven. Here we present a case of paralytic exotropia due to oculomotor nerve palsy on a 32-year-old male along with evidence supporting our decision of surgical management. A 32-year-old male with a chief complaint of squint in both eyes was examined at an outpatient eye clinic. The patient had congenital isolated partial oculomotor nerve palsy and the examination revealed severe ptosis on both eyes with 40 prism diopter exotropia and no limitation in the forced duction test. The patient's posterior segment examination showed normal results, and a diagnosis of bilateral paralytic exotropia due to isolated partial oculomotor nerve palsy was considered. The patient underwent a 7.5 mm rectus lateral recession with a 6 mm rectus medial resection on his right eye, with a promising result after a 3-week follow-up. When treating paralytic strabismus resulting from isolated oculomotor nerve palsy, a combination of medial rectus resection and lateral rectus recession yields good results and long-term stability.

## Introduction

Among the three ocular motor nerve palsies, oculomotor nerve palsy presents the greatest challenge in clinical treatment because it affects four of the six extraocular muscles, necessitating alternative strategies. Ptosis, pupillary involvement, amblyopia, oculomotor synkinesis, bell's phenomenon weakness, superior oblique (SO) overaction, and lateral rectus (LR) contracture are some of the other associated conditions that can worsen the situation further [1].

The oculomotor nerve can be isolated or together with paralysis of other optic nerves. Isolated oculomotor nerve palsy can be congenital or acquired, unilateral or bilateral, total or partial, and with or without pupillary involvement. In children, congenital (43%), trauma (20%), inflammation (13%), aneurysm (7%), and migraine ophthalmoplegia are the most common causes of isolated oculomotor nerve palsy [1]. On the other hand, vasculopathies (such as diabetes mellitus and hypertension), aneurysms, and trauma are common etiologies in the adult population.

Surgery remains the main form of treatment as non-surgical methods are not very helpful [2]. Different surgical techniques are used for oculomotor nerve palsies, both total and partial. One of the most frequently used surgical procedures today is to fix the ocular muscles to the periosteal layer on both the nasal and lateral sides of the orbital bone using a Callahan suture. This technique does show satisfactory results [3]. However, this technique has relatively high procedural difficulties and a high recurrence rate, ranging from 22-59% when performed on patients with exotropia [4]. Along with this fact, there are other surgical techniques that are procedurally simpler with the same or even lower recurrence rate called the recess-resect technique which simplifies the previously complex procedure by eliminating the periosteal suture process. In this paper, we report a case of isolated oculomotor nerve palsy with evidence supporting the technique used to manage this patient.

## Patient and observation

**Patient Information:** a 32-year-old male came to the outpatient eye clinic with a chief complaint of squint in both eyes. The patient felt his squint towards his ears. Any sudden vision loss or double vision was denied. He admitted that his squint was not deteriorating nor improving with any particular activities. He recalls that he has had his squint since he was a kid. Besides that, since he was a baby, he felt that his upper eyelid was always dropping down, and it also did not get heavier with activities. He had never come to an ophthalmologist before. We found no relevant past medical history related to his complaint.

**Figure 1:**
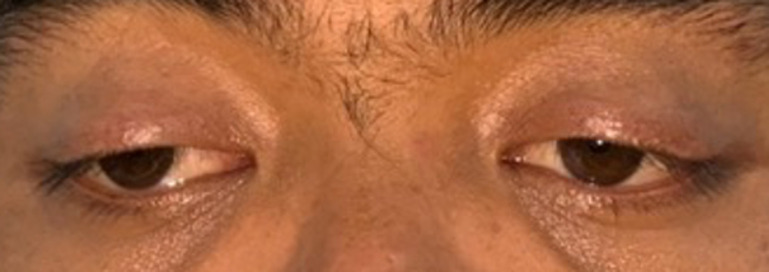
clinical photo of the patient revealing a ptosis on both eyes

**Figure 2:**
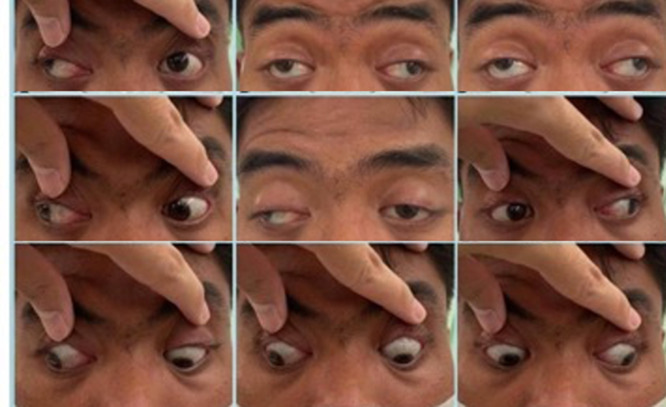
preoperative nine-gaze examination revealing limitation in supraduction and adduction in both eyes

**Clinical findings:** his visual acuity was 5/60 in his RE and it was improving up to 6/6 (with S-4.25 D) and 4/60 in his left eye and was improving to 6/6 (with S-5.75 D) with a normal intraocular pressure. The cover test showed RE exotropia with a positive alternate cover test. We also found severe ptosis on both eyes with measurement no appearing lid crease which indicates a congenital underlying condition. The interpalpebral fissure (FIP) measurement was 7 mm on both eyes, margin to reflex distance 1 (MRD 1) was 1 mm on both eyes, MRD 2 was 6 mm on both eyes, margin limbal distance was 7 mm on both eyes, and his levator action was 6 mm on both eyes (Figure 1). We found limitation in supraduction and adduction in ocular motility assessment (Figure 2).

**Diagnostic assessment:** Hirschberg test showed 30° exotropia with 40 Prism Diopter (PD) Base In (BI). We also found no limitation in the forced duction test in both eyes. Posterior segment examination showed normal results.

**Diagnosis:** bilateral paralytic exotropia due to isolated partial oculomotor nerve palsy.

**Figure 3:**
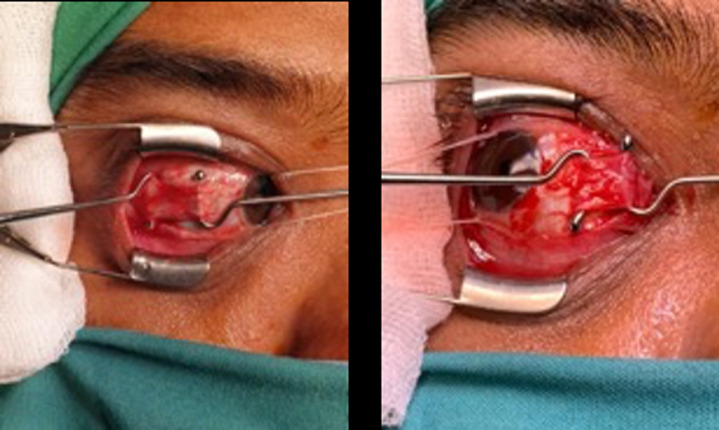
clinical photos of the lateral rectus muscle and medial rectus muscle

**Therapeutic interventions:** the patient underwent a 7.5 mm lateral rectus recession and 6 mm medial rectus resection on his right eye (Figure 3).

**Figure 4:**
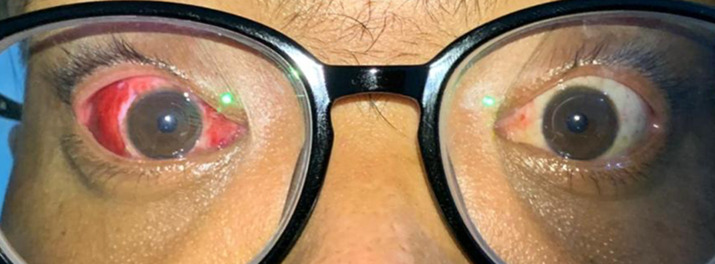
primary gaze day one after surgery

**Figure 5:**
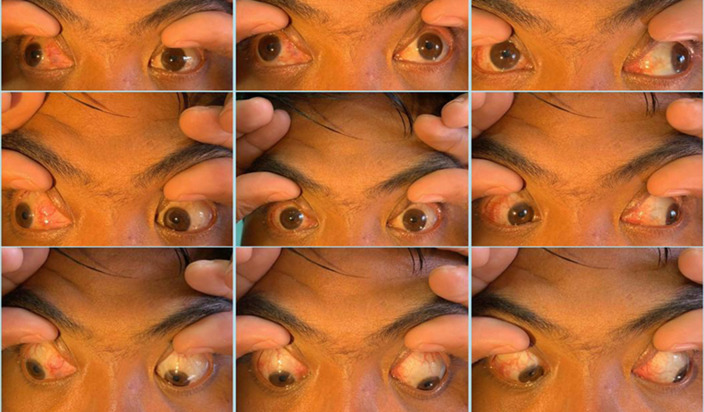
improvement of ocular motility in 3-weeks post operation

**Follow-up and outcome of interventions:** immediate postoperative examination showed a promising result (Figure 4) proven with a 3-week follow-up examination showing improvement in his ocular motility (Figure 5).

**Patient perspective:** the patient was very satisfied with the surgery result and it would definitely increase his self-confidence.

**Informed consent:** patient and family were informed about the case reported and they gave their consent for its publication for the benefit of people and medicine.

## Discussion

Among the therapies for paralytic strabismus, surgery for paralytic exotropia is thought to be the most challenging. Transposition of the vertical oblique muscle produces very good results in situations with full palsy. The medial rectus muscle can be excised in cases of partial palsy. When combined with lateral rectus muscle recession, these techniques are more effective. Whether or not the medial rectus muscle is resected, the outcomes of transposing the superior oblique muscle are identical. Currently published evidence showed that combining recession and resection procedures in the management of paralytic strabismus showed a comparable, if not better, result with a procedure involving fixing the ocular muscles to the periosteal layer on both the nasal and lateral sides of the orbital bone using a Callahan suture [5-8].

It is challenging to determine the etiology of the oculomotor nerve palsy in our patient considering the unusual presentation especially the fact that his complaint has been felt since he was a kid which introduced the potential for recall bias in the history-taking process of our diagnosis. However, based on our data, congenital oculomotor nerve palsy along with neurogenic ptosis was the main suspicion underlying his complaint. As reported by Priya *et al*. in 2021, isolated oculomotor nerve palsy was mainly congenital in nature rather than acquired [1,9].

Despite the uncertainty of etiology, our decision to manage the patient with a combined recession and resection procedure was proven to be beneficial to the patient's complaint, even after 3 weeks of follow-up. The consideration of only operating on the right eye was mainly regarding the most dominant deviated eye. The non-operated eye is still intact and hopefully will not undergo further surgery if there are any post-operative complications. Our technique for figuring out how much recession versus resection to do is still being refined; at this point, evidence both published and unpublished in our healthcare facility is still scarce to create an adequate and rigid formula.

## Conclusion

Combining lateral rectus recession and medial rectus resection is effective for treating paralytic strabismus due to isolated oculomotor nerve palsy with good outcomes. This procedure is a win-win solution for both the patient and the surgeon as this procedure was less complex than the widely used procedure of periosteal fixation with Callahan suture.
